# All are equal, but some are more equal than others: social determinants of leisure time physical activity through the lens of intersectionality

**DOI:** 10.1186/s12889-021-12428-7

**Published:** 2022-01-06

**Authors:** Gregore I. Mielke, Deborah C. Malta, Bruno P. Nunes, John Cairney

**Affiliations:** 1 School of Human Movement and Nutrition Sciences, The University of Queensland, Brisbane, Australia; 2 Programa de Pós-graduação em Saúde Pública, Faculdade de Medicina. Universidade Federal de Minas Gerais, Av. Alfredo Balena 190, Santa Efigênia, Belo Horizonte, MG 30130-100 Brazil; 3 Department of Nursing in Public Health, Universidade Federal de Pelotas, Pelotas, Brazil

**Keywords:** Physical activity, Social determinants, Epidemiology, Population health, Adults

## Abstract

**Background:**

To date, no research has investigated social determinants of leisure time physical activity through the lens of intersectionality in a low- and middle-income country. Therefore, the aim of this study was to explore the intersectionality in leisure time physical activity in a nationwide sample of Brazilian adults.

**Methods:**

Data from the Brazilian National Health Survey conducted in 2013 were analysed (*N* = 58,429). Prevalence of sufficient leisure time physical activity (150+ minutes per week in moderate-to-vigorous physical activity) was estimated according to gender, racial identity, education and income, and according to multiple combinations of these sociodemographic characteristics (i.e., multiple jeopardy index).

**Results:**

The prevalence of sufficient leisure time physical activity was 22.9% (95%CI: 22.3 to 23.6). Overall, the prevalence of sufficient leisure time physical activity was highest among men, individuals with white skin colour, and among those in the highest group of education and income. Among men, white, with a university degree and in the highest quartile of income (3% of the population), the prevalence of sufficient leisure time physical activity was 48%. Among non-white women with low education and low income (8.1% of the population), the prevalence of sufficient leisure time physical activity was 9.8%.

**Conclusion:**

Informed by the theory of intersectionality, findings of this study have shown that intersections of gender, racial identity and socioeconomic position of the Brazilian society strongly influence leisure time physical activity at the individual level. Targeted interventions to increase leisure time physical activity should address the complexities of social status intersections.

## Background

In continents such as North America, South America, Western Europe, and Australasia, epidemiological research has documented consistent social status differences in physical activity behaviours [1]. Women [2], those with less education and low income [3], older adults and individuals from visible minority groups [1] have been shown to be less physically active than relatively advantage social groups (e.g., men, white, higher SES). These same disadvantaged social positions are also related to a variety of morbid conditions and mortality – collectively described as the social determinants of disease [4–6].

The vast majority of research in physical activity epidemiology has employed what social scientists have called a monistic theory of social inequality [7], meaning researchers have tended to privilege one particular marker of social disadvantage (e.g., gender or social class) over others. For example, researchers interested in gender differences in physical activity behaviours focus on both the main effect (gender differences) and adjusted effect, with the assumption of no interaction (gender difference after statistically controlling for other variables related to the physical activity) to understand the impact of one specific marker of social disadvantage, as though it were largely independent of the influence of other social determinants. Such an approach is consistent with social theories such as feminism or Marxism, which also view a particular form of social inequality as dominant over others (e.g., gender and social class respectively) [7].

An alternative theory is intersectionality, which has its roots in feminism and qualitative research [8]. Here researchers both acknowledge and examine how multiple, intersecting social statuses create more complex social hierarchies, which are defined by multiple and intersecting systems of social disadvantage or oppression [9]. In health inequality research, researchers have documented a multiple jeopardy effect, showing, for example, that gender, race and socioeconomic status work multiplicatively to produce poor health [10, 11]. It is not the additive effects of being poor, non-white and a woman, but the combined synergistic effect of these statuses that together influence health inequalities.

Intersectionality approaches to the study of social determinants of physical activity behaviours, however, has been slow in adoption among physical activity epidemiologists. Using national health surveillance data from Canada, Abichahine and Veenstra (2016) [12] found that in two nationally representative samples of Canadians, the positive effect of income on recreational, leisure based physical activity was strongest among visible minority males, moderately related among white men and women, but had no effect among visible minority women. Being lesbian, gay or bisexual was associated with higher levels of physical activity participation for women, especially visible minority women, but not men [12].

Qualitative research has similarly documented intersections of social disadvantage and the impact this has on physical activity. Roberts et al. (2019) [13] found race and social class to be associated with active transport behaviours in adolescent, though the impact of both was more pronounced in girls compared with boys. Ray (2014) [14] explored the intersections of race, place, gender and body convergence to shape physical activity behaviour in middle class Black women, in ways that are different from other race and gendered social groups. Cairney and colleagues (2015) examined the interactive effects of neighbourhood income, gender (sex) and aging on participation in sport and free play (unorganized active play) in children as they aged from peri- to mid-adolescence [15]. Their work revealed that while the effect of income on sport remained constant over time for both boys and girls (higher income associated with greater participation), for girls, income inequality in free play widened over time. This study suggests not only age, gender and income differences are important, but the type of participation may also be influenced by different combinations of social determinants.

Together, while this small body of work suggests the importance of considering how intersecting social statuses influence physical activity behaviour, important knowledge gaps exist. Understanding how physical inactivity is structured by intersecting positions of social status is important for the effective design of population level prevention strategies, allowing for the tailoring of education and behavioural change programs to specific, vulnerable subgroups in the population. Moreover, intersecting social structures may vary according to country income and macro level social inequalities, which is also likely to have influence on different domains of physical activity, such as leisure, transportation and occupation. Furthermore, understanding social determinants of leisure time physical activity through the lens of intersectionality is important because leisure time physical activity is a domain with the utmost potential for public health interventions, and it is likely to provide more benefits for health than other domains of physical activity such as occupational physical activity [16, 17].

For example, in Brazil, the 6th most populous country in the world and ranked among the highest countries in the world in terms of social inequality [18], data from national health surveys have suggested that, whereas education is not associated with leisure time physical activity, higher income is strongly associated with high levels of leisure time physical activity [3], a pattern which has not been observed in high-income countries [1]. In Brazil, education is also an important correlate of leisure time physical activity during and post pregnancy in women [19]. Moreover, socioeconomic inequalities identified in men and women of different age groups also vary according to regional areas [20]. Up to date, no research has investigated social determinants of leisure time physical activity through the lens of intersectionality in a low- and middle-income country. Therefore, the aim of this study was to explore the intersectionality in leisure time physical activity in a nationwide sample of Brazilian adults.

## Methods

### Design and sample

We used data from the Brazilian National Health Survey conducted in 2013. This was a home-based survey conducted by the Brazilian Institute of Geography and Statistics in collaboration with the Ministry of Health [21]. The sampling process was built in order to obtain a representative sample of the Brazilian population. The sampling process was carried out in three stages, with census sectors as the primary sampling units, households as the secondary units, and adults (18 years or older) residents of the households as the tertiary sampling units. In each household sampled, one adult was selected equiprobabilistically. Each participant responded to one questionnaire which included household information, and one questionnaire about lifestyle and health conditions. Additional details about the National Health Survey have been published elsewhere [21]. The study was approved by the National Commission for Research Ethics (No. 328.159, 26 June 2013). All individual participants were consulted, clarified and accepted participation in the study by signing of an Informed Consent Form.

### Leisure time physical activity

Leisure time physical activity was measured using a questionnaire that collected information on frequency and duration of multiple leisure time physical activities. The following questions were used: *“1) Have you participated in any type of physical activity or sport in the last three months?; 2) what was the main type of physical activity or sport that you participated?; 3) Do you exercise at least once a week?; 4) How many days per week do you usually do physical activities or sports?; 5) On these days, how long do these activities last?”*. This questionnaire has been used in the Risk and Protection Factors Surveillance system for Chronic Diseases by Telephone Survey (VIGITEL) since 2006 [20, 22], and has demonstrated to be reliable and valid for assessing leisure time physical activity [Kappa coefficient: 0.70 (95% CI: 0.62–0.78)] in Brazilian adults [23].

A weekly score of physical activity was created, multiplying the time spent in leisure time physical activities by the number of days per week. Participants who reported vigorous activities (running, aerobics/spinning/step/jump, football, basketball or tennis) had their time multiplied by two to match with current physical activity guidelines (150 min per week of moderate intensity physical activity, or 75 min in vigorous intensity physical activity, or a combination of both) [24]. Participants who reported at least 150 min per week of moderate to vigorous intensity leisure time physical activity were classified as active [24] (henceforth referred to as ‘prevalence of leisure time physical activity’).

### Sociodemographic variables

Questionnaires administered by interviewers were used in the National Health Survey to collect sociodemographic data from participants. This included gender (men, women), age (categorised in 18–24, 25–34, 35–74, 75+ years), racial identity (respondents were asked to self-declare their race/skin-color as white, black, brown, yellow and native indigenous; for the analyses participants were categorised as white or non-white) [25], highest level of formal education (categorised in four levels as none or incomplete primary, complete primary or incomplete secondary, complete secondary or incomplete university, university graduate). Participants reported their total family income, which was categorised in quartiles.

### Social jeopardy index

Our analyses were based on the principle of “multiple jeopardy”, one of the principles that serve to guide investigations about intersectionality theory and social phenomena. In summary, multiple jeopardy is the theory that the various factors of one’s identity that led to discrimination or oppression, such as gender, class, or race are interdependent and have a compounded or cumulative effect on the discrimination that person experiences [26]. Therefore, we created a composite score by assigning the most privileged group of each variable a score of zero (men, white, highest education, and highest family income) and the least privileged group a score of one (women and non-white) or three (none or incomplete primary level of education and the lowest quartile of family income). Therefore, for each variable the following scores were assigned: gender (men = 0; women = 1); racial identity (white = 0; non-white = 1); education (university graduate = 0; complete secondary or incomplete university = 1; complete primary or incomplete secondary = 2; none or incomplete primary = 3); family income (top quartile = 0; 3rd quartile = 1; 2nd quartile = 2, bottom quartile = 3). Scores for each indicator were summed, resulting a ‘Jeopardy Index’ ranging from 0 to 8, where the lowest group (score = 0) included men, white and university degree and in the highest quartile of income, whereas the highest score (score = 8) was composed by women, non-white, with none or incomplete primary level of education, and in the bottom quartile of income.

### Statistical analysis

To explore the relationship between individual and combined sociodemographic variables, the analyses were performed in five steps. First, descriptive analyses were conducted to describe the prevalence of leisure time physical activity according to each individual sociodemographic characteristic of the sample. Second, the association between each sociodemographic variable and leisure time physical activity was investigated using a series of Poisson regressions. Unadjusted and adjusted analyses were performed, with simultaneous adjustment for each sociodemographic variable. Third, the association between the Jeopardy Index and leisure time physical activity was investigated. The prevalence of leisure time physical activity was calculated for each level of the Jeopardy Index. Additionally, we used a graphical representation based on the Lorenz curve [27] to demonstrate population inequalities in leisure time physical activity according to Jeopardy Index. For this, cumulative percentage of population by Jeopardy Index (x axis) were plotted against the cumulative distribution of leisure time physical activity in the sample (y axis). The graph shows, for example that an x-value of 50 and a y-value of 75 would mean that the bottom 50% of the population in terms of Jeopardy Index has 75% of the total individuals who are activity in that sample. Fourth, we estimated the prevalence of leisure time physical activity for each group based on the combination of sociodemographic characteristics. For this we conducted a logistic regression analysis in which sociodemographic and multiple interaction terms (e.g. gender x racial identity x education x income) were included as predictors of leisure time physical activity. Predicted prevalence of leisure time physical activity was calculated and graphically present with respective 95% confidence intervals (95%CI). Finally, we also estimated the prevalence of leisure time physical activity for each score of the Jeopardy Index according to age groups. This strategy was used to exemplify the extent to which effects of multiple social jeopardy (social determinants of health) are comparable with the well know effects of age on leisure time physical activity. In all analyses sample weighting and the complexity of sample selection were taken in account. Data were interpreted based on 95% confidence intervals. Statistical analyses were performed using Stata, version 16.

## Results

Of 81,167 individuals who were sampled across Brazil, 60,202 were interviewed and 58,429 provided information about leisure time physical activity. These included women (53%), non-white (51%), with incomplete primary education (39%). Nearly 16 and 5% were aged 18–24 and 75y+, respectively (Table 1). The estimates of this sample can be extrapolated to over 141 million Brazilian adults.

**Table 1:** Sociodemographic characteristics and prevalence of leisure time physical activity according to sociodemographic variables. Brazil 2013. *N* = 58,429*

Variables	N (weighted %)	LTPA % (95%CI)	***p*** value
Gender			< 0.001
Men	25,007 (46.9)	27.7 (26.7–28.7)	
Women	33,434 (53.1)	18.7 (17.9–19.3)	
Skin colour			< 0.001
White	23,934 (48.5)	24.1 (23.2–25.1)	
Non-white	34,504 (51.4)	21.8 (21.0–22.6)	
Educational level (grade)			< 0.001
University degree	7584 (12.8)	38.4 (36.4–40.4)	
High School	18,645 (32.9)	29.4 (28.2–30.6)	
Primary education	8947 (15.5)	24.1 (22.5–25.8)	
Incomplete primary education	23,265 (38.7)	11.8 (11.1–12.6)	
Income (quartiles)			< 0.001
Q4 (highest)	13,031 (23.2)	31.9 (30.5–33.3)	
Q2	16,177 (28.4)	22.8 (21.7–24.0)	
Q2	5700 (8.9)	19.5 (17.6–21.7)	
Q1 (lowest)	23,533 (39.4)	18.5 (17.6–19.5)	
Age groups			< 0.001
18–24	7554 (15.8)	36.3 (34.3–38.3)	
25–34	13,482 (25.6)	27.6 (26.3–29.0)	
35–74	34,584 (58.0)	18.7 (18.0–19.5)	
75+	2821 (4.6)	8.1 (6.6–9.9)	

*extended *N* = 141,709,170 inhabitants

The prevalence of leisure time physical activity was 22.9% (95%CI: 22.3 to 23.6). Overall, the prevalence of leisure time physical activity was highest among men, individuals with white skin colour, and among those in the highest group of education and income (Table 1). In the crude analyses, the prevalence of leisure time physical activity was 32% lower in women than in men, and 15% lower in non-white than white individuals (Table 2). A strong inverse association of education and income with leisure time physical activity was observed. Compared with those in the highest group of education and income, those in the bottom groups were, respectively, 69 and 42%, less likely to meet current physical activity guidelines during leisure time. In the mutually adjusted analyses, only gender and education remained associated white leisure time physical activity (Table 2).

**Table 2:** Crude and adjusted association between sociodemographic variables and leisure-time physical activity (*N* = 58,429)

Variables	Crude	Mutually adjusted
	Prevalence ratio (95%CI)	Prevalence ratio (95%CI)
Gender		
Men	1.00	1.00
Women	0.68 (0.64–0.71)	0.66 (0.62–0.69)
Skin colour		
White	1.00	1.00
Non-white	0.90 (0.86–0.95)	1.04 (0.99–1.10)
Educational level (grade)		
University degree	1.00	1.00
High School	0.76 (0.72–0.82)	0.75 (0.70–0.80)
Primary education	0.63 (0.58–0.68)	0.60 (0.55–0.66)
Incomplete primary education	0.31 (0.28–0.34)	0.30 (0.27–0.33)
Income (quartiles)		
Q4 (highest)	1.00	1.00
Q2	0.72 (0.67–0.77)	0.96 (0.89–1.03)
Q2	0.61 (0.55–0.69)	1.07 (0.95–1.20)
Q1 (lowest)	0.58 (0.54–0.62)	0.99 (0.89–1.07)

**Fig. 1:**
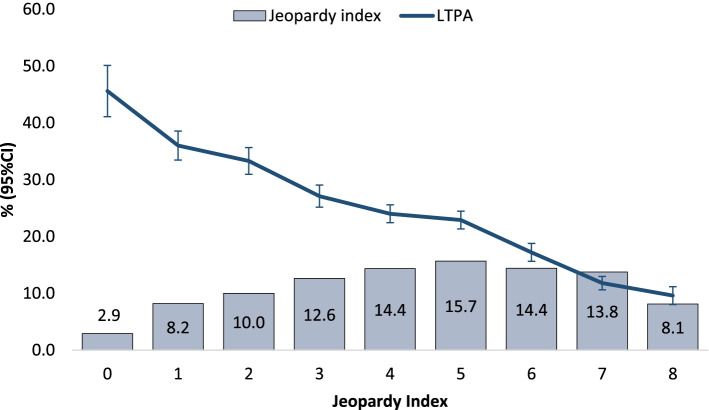
Distribution of Jeopardy Index and observed prevalence of leisure time physical activity according to Jeopardy Index (*N* = 58,429). Brazil, 2013

Overall, the jeopardy index was inversely associated with the prevalence of leisure time physical activity. As demonstrated in Fig. 1, nearly 3% of the population were men, white, with university degree and in the highest quartile of income. Among this group, the prevalence of physical activity was 48%, whereas among non-white women with low education and low income (8.1% of the population), the prevalence of leisure time physical activity was 9.8%.

**Fig. 2:**
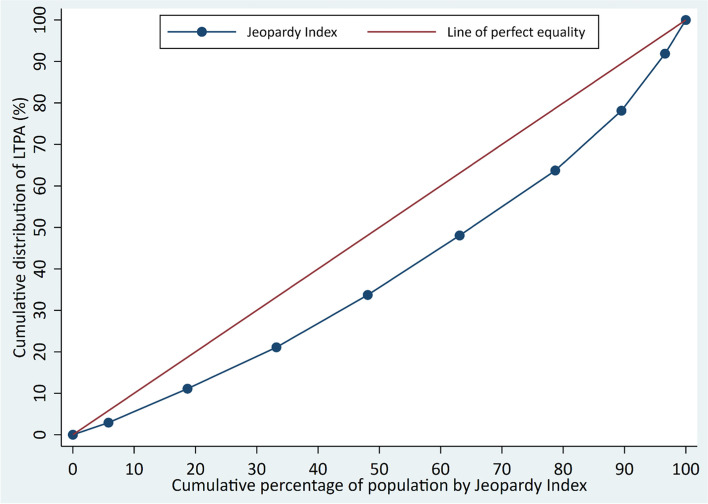
Lorenz curve to demonstrate inequalities in LTPA (*N* = 58,429). Brazil, 2013

Inequalities in leisure time physical activity according to the Jeopardy Index are further presented in the Lorenz curve in Fig. 2. The graph shows, that 3% of the population with a Jeopardy Index of 0 included 6% of active population. Moreover, 48% of population with a Jeopardy Index lower than 5 had 63% of the active population.

**Fig. 3:**
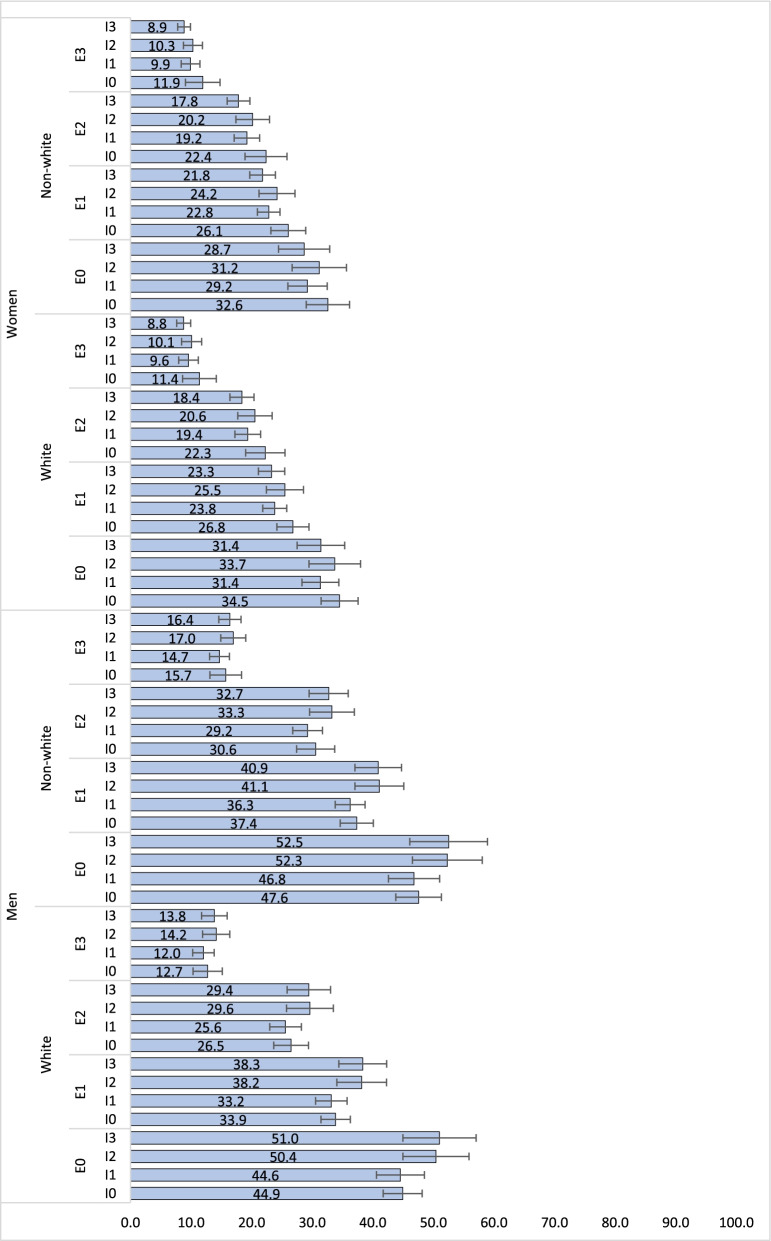
Predicted prevalence of leisure time physical activity according to groups [E0: university degree; E3: Incomplete primary education; I0: highest quartile of income; I3: lowest quartile of income]. Predicted probabilities were estimated using logistic regression models which included multiple interaction for pair of sociodemographic variables (i.e., gender x skin-colour; gender x education; gender x income; skin-colour x education; skin-colour x income; education x income)

Estimates of the prevalence of leisure time physical activity according to multiple sociodemographic groups are presented in Fig. 3. Although the highest prevalence of leisure time physical activity was observed among men, non-white with university degree education and in the lowest quartile of income, the confidence intervals of the prevalence of leisure time physical activity for this sub-group overlapped with estimates of the prevalence of leisure time physical activity observed in other sub-groups, particularly those with similar level of formal education. Among women, both education and income were associated with higher prevalence of leisure time physical activity. However, among men, the prevalence of leisure time physical activity increased with education, but not with income. Among non-white men with at least higher education, the prevalence of leisure time physical activity was inversely associated with quartiles of income.

**Fig. 4:**
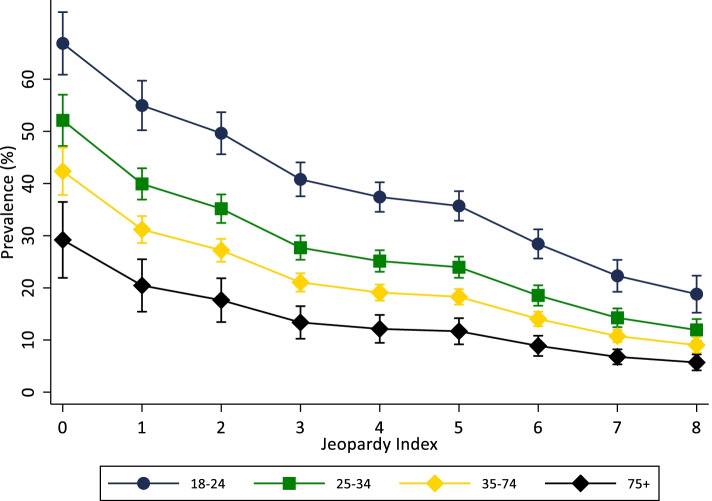
Predicted prevalence of leisure time physical activity according to Jeopardy Index and age groups. Brazilian National Health Survey, 2013. (*N* = 58,429)

Figure 4 is used to demonstrate the extent to which effects of multiple social jeopardy (social determinants of health) are moderated by age, and comparable with the effects of age on leisure time physical activity. Overall, each increment of one point in the Jeopardy Index was associated with 16% lower prevalence of leisure time physical activity (Prevalence ratio 0.84; 95%CI: 0.83 to 0.84; p for linear trend < 0.001). However, this linear decrease varied from 12% (Prevalence ratio 0.88; 95%CI: 0.85 to 0.90; p for linear trend < 0.001) in individuals 18–24-year-old to 21% (Prevalence ratio 0.79; 95%CI: 0.72 to 0.88; p for linear trend < 0.001) among those 75y + older (Fig. 4).

As demonstrated in Fig. 4, among individuals with a Jeopardy Index of zero (men, white, with university degree and high income) the prevalence of leisure time physical activity was 69% lower in the oldest age group (75y+) than in the youngest group (18-24y). Moreover, the predicted prevalence of leisure time physical activity among adults 18–24 years old with the highest score of the Jeopardy Index (18.8%; CI95%: 15.2 to 22.4%) was lower than the prevalence of leisure time physical activity in older people (75y+) with the lowest score of the Jeopardy Index score (29.2%; 95%CI: 21.9 to 36.5%).

## Discussion

In this study we explored the role of intersectionality on leisure time physical activity in a large sample of Brazilian adults. Our findings have shown that only one in four adults meet the current physical activity guidelines during leisure time, with this estimate varying from 10 to 75% depending on the sociodemographic characteristics of population groups. Informed by intersectionality theory, this study was the first to examine whether gender, racial identity, and socioeconomic position ‘intersect’ with one another to predict leisure time physical activity in a large middle-income country with 212 million inhabitants. These findings highlight the challenge and complex pathway to be faced if we aim to increase population levels of leisure time physical activity.

The large variation in the prevalence of leisure time physical activity by gender has been extensively reported in the literature [1, 2, 28]. In our study, women were less active during leisure time than men. Recent studies have shown that the relationship between gender and physical activity depends on the domain of physical activity [3, 28, 29]. In Brazil, an abundance of evidence shows that men are more active than women, and that education and income are positively associated with leisure time physical activity [3, 20, 22, 30].

Our findings based on the extent to which gender, racial identity, and socioeconomic position have a cumulative effect to determine leisure time physical activity reinforce that leisure time physical activity is likely to be another privilege of well-educated, wealthy and withe men [31]. This social privilege might be explained by the availability and more access to private leisure time physical activity facilities, access to green areas, flexibility in work arrangements, access to childcare facilities, social norms, psychosocial factors, and participation in other domains of physical activity, which largely vary by sociodemographic groups and are important correlates of leisure time physical activity [1]. Moreover, at a macro-level, the social privilege is also explained by the concentration of power that economic elites hold, and the institutionalised sexism and racism permeated by historical and cultural characteristics of the Brazilian society [32].

Previous studies that investigated ‘independent effects’ of gender and social inequalities in leisure time physical activity used multivariate regression models with mutual adjustment for other factors [3, 20, 33]. While this approach has provided important insights, they fail to consider the social complexities within societies and the connections between macro-level structures of power on the creation, maintenance and reproduction of social inequalities at lower level analyses [34].

It is not new in the scientific literature that younger and more educated individuals are more physically active during leisure time than their peers [1, 35–37]. Findings from our study have shown that the potential effects of multiple jeopardy on leisure time physical activity might be comparable to the well-known age-effect on leisure time physical activity. By comparing the prevalence of leisure time physical activity among adults 18–24 years old in the highest score of the jeopardy index with the prevalence of leisure time physical activity in older people (75y+) in the lowest score of the jeopardy index score, data from this study suggest that social inequalities might ‘age’ women, non-white, with low education and low income up to 50 years.

Recognition of systemic and intersecting inequities in physical activity begins by recognising that leisure time physical activity is likely to be another privilege for some population groups [31]. As demonstrated by our data, men with low education might experience disadvantage in physical activity over women with high education, despite the prevailing gender differences in leisure time physical activity. Our data also reinforce that neither educated women nor low educated men are homogeneous in terms of leisure time physical activity, which is likely to be a product of different opportunities and challenges faced by each group. Thus, the recognition of leisure time physical activity as privilege and not merely as a choice in the Brazilian context (and probably in other low-middle income countries) is important because, according to Knuth and Antunes (2021) [31], by assuming that privileges are strong determinants of participation in leisure time physical activity, we place ourselves as advocates of public health policies that address major social challenges, such as poor living conditions, lack of access to quality education and health services, and inadequate working conditions [38]. Moreover, given that the health benefits of physical activity are likely to be stronger for leisure time physical activities than for other domains of physical activity, particularly occupational physical activity [16], understanding the major social determinants of leisure time physical activity is imperative for implementation of interventions aimed to improve access to leisure time physical activity programs for population groups that historically have been left behind in terms of public health interventions.

Despite advances in the recent decades in the confrontation of socioeconomic inequalities, Brazil still faces large social inequalities [18, 39]. The existing inequalities are an important challenge because they are strongly related to aspects that increase chances of individuals to access leisure time physical activity facilities, which, in Brazil, are still mostly private spaces. This is evidenced by previous studies which showed that lack of money was twice as often reported as a barrier to participation in leisure time physical activity in individuals with low socioeconomic position than among those with high socioeconomic position, yet disliking physical activity was reported in a similar proportion between low and high socioeconomic groups [40].

Our findings suggests that promotion of leisure time physical activity in Brazil should address inequalities and be shaped by the intersection of gender, race and socioeconomic position. In 2006, the Brazilian Ministry of Health launched the National Health Promotion Policy [38], which led to the *Academia da Saúde Program* (Health Academy in plain translation) in 2011. Throughout this initiative, over 2700 municipalities in Brazil received federal funding to development community-based interventions to enhance access of the population to health professionals and facilities for participation in leisure time physical activity [38, 41]. However, despite numerous evaluations of the *Academia da Saúde Program* have demonstrated the potential of this initiative to foster social inclusion and democratise access to leisure time physical activity, especially for women, older and individuals with low socioeconomic position [30, 38, 42], current policies of austerity that are in place in Brazil are a constant threat to the consolidation of this program [43, 44].

Limitations of this study must be acknowledged. First, although physical activity is a multi-domain behaviour that can occur at work, domestic activities and commuting, our analyses were restricted to the leisure time because we believe that this domain has the utmost potential for intervention. Future studies should further investigate disparities and intersectionality in relation to other domains of physical activity. The self-reported measure of physical activity and the operational definition used in this study might overestimate individual levels of leisure time physical activity. Moreover, the sociodemographic indicators used in these analyses are still likely to reflect a simplistic view of the far more complex social intersections that affect leisure time physical activity. However, we believe that the inclusion of gender, racial identity, education and income can be an important indicator of the hierarchical structures existent in Brazil.

## Conclusion

Informed by the principles of intersectionality, our study has the potential to initiate discussions about the complex nature of social inequality in leisure time physical activity in Brazil. Findings of this study have shown that intersections of gender, racial identity and socioeconomic position at macro-levels observed in the Brazilian society, strongly influence leisure time physical activity at the individual level. Therefore, public health interventions designed to increase populations levels of leisure time physical activity should take into account the complexities of social status intersections.

## Data Availability

The datasets used and/or analysed during the current study are available from the corresponding author on reasonable request.

## References

[1] Bauman AE, Reis RS, Sallis JF, Wells JC, Loos RJ, Martin BW (2012). Correlates of physical activity: why are some people physically active and others not?. Lancet..

[2] Mielke GI, da Silva ICM, Kolbe-Alexander TL, Brown WJ (2018). Shifting the physical inactivity curve worldwide by closing the gender gap. Sports Med.

[3] da Silva ICM, Mielke GI, Bertoldi AD, Arrais PSD, Luiza VL, Mengue SS (2018). Overall and leisure-time physical activity among Brazilian adults: National Survey Based on the global physical activity questionnaire. J Phys Act Health.

[4] Syme SL (1987). Social determinants of disease. Ann Clin Res.

[5] Syme SL (2005). Historical perspective: the social determinants of disease - some roots of the movement. Epidemiol Perspect Innov.

[6] Cockerham WC, Hamby BW, Oates GR (2017). The social determinants of chronic disease. Am J Prev Med.

[7] Fox NJ, Alldred P (2018). Social structures, power and resistance in monist sociology: (new) materialist insights. J Sociol (Melb).

[8] Cooper B, Disch L, Hawkesworth M (2015). Intersectionality. The Oxford Handbook of Feminist Theory.

[9] McMullin JA, Cairney J (2004). Self-esteem and the intersection of age, class, and gender. J Aging Stud.

[10] Ferraro KF, Farmer MM (1996). Double jeopardy to health hypothesis for African Americans: analysis and critique. J Health Soc Behav.

[11] Taylor D, Richards D (2019). Triple jeopardy: complexities of racism, sexism, and ageism on the experiences of mental health stigma among young Canadian black women of Caribbean descent. Front Sociol.

[12] Abichahine H, Veenstra G (2017). Inter-categorical intersectionality and leisure-based physical activity in Canada. Health Promot Int.

[13] Roberts JD, Mandic S, Fryer CS, Brachman ML, Ray R. Between Privilege and Oppression: An Intersectional Analysis of Active Transportation Experiences Among Washington D.C. Area Youth. Int J Environ Res Public Health. 2019;16(8):1313. 10.3390/ijerph16081313. 10.3390/ijerph16081313 PMC6518066 31013698

[14] Ray R. An Intersectional analysis to explaining a lack of physical activity among middle class black women. Race Ethn. 2014. 10.1111/soc4.12172.

[15] Cairney J, Joshi D, Kwan M, Hay J, Faught B (2015). Children's participation in organized sport and physical activities and active free play: exploring the impact of time, gender and neighbourhood household income using longitudinal data. Sociol Sport J.

[16] Holtermann A, Hansen JV, Burr H, Sogaard K, Sjogaard G (2012). The health paradox of occupational and leisure-time physical activity. Br J Sports Med.

[17] Moore SC, Lee IM, Weiderpass E, Campbell PT, Sampson JN, Kitahara CM (2016). Association of Leisure-Time Physical Activity with Risk of 26 types of Cancer in 1.44 million adults. JAMA. Intern Med.

[18] Bank W (2019). World Bank Open Data.

[19] Mielke GI, Crochemore-Silva I, Domingues MR, Silveira MF, Bertoldi AD, Brown WJ (2021). Physical activity and sitting time from 16 to 24 weeks of pregnancy to 12, 24, and 48 months postpartum: findings from the 2015 Pelotas (Brazil) birth cohort study. J Phys Act Health.

[20] Mielke GI, Malta DC, de Sa GB, Reis RS, Hallal PC (2015). Regional differences and correlates of leisure time physical activity in Brazil: results from the Brazilian National Health Survey-2013. Rev Bras Epidemiol.

[21] Szwarcwald CL, Malta DC, Pereira CA, Vieira ML, Conde WL, Souza Junior PR (2014). National Health Survey in Brazil: design and methodology of application. Cien Saude Colet.

[22] Mielke GI, Hallal PC, Malta DC, Lee IM (2014). Time trends of physical activity and television viewing time in Brazil: 2006-2012. Int J Behav Nutr Phys Act.

[23] Moreira AD, Claro RM, Felisbino-Mendes MS, Velasquez-Melendez G (2017). Validity and reliability of a telephone survey of physical activity in Brazil. Rev Bras Epidemiol.

[24] Bull FC, Al-Ansari SS, Biddle S, Borodulin K, Buman MP, Cardon G (2020). World Health Organization 2020 guidelines on physical activity and sedentary behaviour. Br J Sports Med.

[25] Kabad JF, Bastos JL, Santos RV. Race, Color and ethnicity in epidemiologic studies carried out with Brazilian populations: systematic review on the PubMed database. Physis. 2012;22(3).

[26] King DK. Multiple jeopardy, multiple consciousness: the context of a black feminist ideology. Signs J Women Cult Soc. 1998;14(1).

[27] Lorenz M (1905). Methods of measuring the concentration of wealth. Publ Am Stat Assoc.

[28] Strain T, Wijndaele K, Garcia L, Cowan M, Guthold R, Brage S (2020). Levels of domain-specific physical activity at work, in the household, for travel and for leisure among 327 789 adults from 104 countries. Br J Sports Med.

[29] Prince SA, Reed JL, Martinello N, Adamo KB, Fodor JG, Hiremath S (2016). Why are adult women physically active? A systematic review of prospective cohort studies to identify intrapersonal, social environmental and physical environmental determinants. Obes Rev.

[30] Simoes EJ, Hallal PC, Siqueira FV, Schmaltz C, Menor D, Malta DC (2017). Effectiveness of a scaled up physical activity intervention in Brazil: a natural experiment. Prev Med.

[31] Knuth AG, Antunes PC. Bodily practices/physical activities considered as privilege and not a choice: analysis in the light of Brazilian inequalities. Saúde Soc. 2021;30(2). 10.1590/S0104-12902021200363.

[32] Souza J (2017). A elite do atraso: da escravidão à Lava-Jato.

[33] Brazo-Sayavera J, Mielke GI, Olivares PR, Jahnecka L, Crochemore MSI. Descriptive epidemiology of Uruguayan Adults' leisure time physical activity. Int J Environ Res Public Health. 2018;15(7). 10.3390/ijerph15071387 PMC6068702 30004431

[34] Dhamoon RK, Hankivsky O, Hankivsky O (2011). Why the Theory and Practice of Intersectionality Matter to Health Research and Policy. Health inequities in canada: intersectional frameworks and practices.

[35] Althoff T, Sosic R, Hicks JL, King AC, Delp SL, Leskovec J (2017). Large-scale physical activity data reveal worldwide activity inequality. Nature..

[36] Hallal PC, Andersen LB, Bull FC, Guthold R, Haskell W, Ekelund U (2012). Global physical activity levels: surveillance progress, pitfalls, and prospects. Lancet..

[37] Sallis JF, Bull F, Guthold R, Heath GW, Inoue S, Kelly P (2016). Progress in physical activity over the Olympic quadrennium. Lancet..

[38] Malta DC, Barbosa da Silva J (2012). Policies to promote physical activity in Brazil. Lancet..

[39] Bertoldi AD, Barros FC, Hallal PRC, Mielke GI, Oliveira PD, Maia MFS (2019). Trends and inequalities in maternal and child health in a Brazilian city: methodology and sociodemographic description of four population-based birth cohort studies, 1982-2015. Int J Epidemiol.

[40] Reichert FF, Barros AJ, Domingues MR, Hallal PC (2007). The role of perceived personal barriers to engagement in leisure-time physical activity. Am J Public Health.

[41] da Saúde M (2017). Panorama Nacional de Implementação do Programa Academia da Saúde. Monitoramento nacional da gestão do Programa Academia da Saúde.

[42] Faria TMTR, Brenner S, Deckert A, Florindo AA, Mielke GI. Health academy program and physical activity levels in Brazilian state capitals. Rev Bras Atividade Fís Saúde. 2020;25. 10.12820/rbafs.25e0133.

[43] Mielke G, Malta DC. Avaliação e futuro do Programa Academia da Saúde. Rev Bras Atividade Fís Saúde. 2020;25.

[44] Brasil. Emenda Constitucional n° 95, de 15 de dezembro de 2016. Altera o Ato das Disposições Constitucionais Transitórias, para instituir o Novo Regime Fiscal, e dá outras providências. Diário Oficial da União 2016; 15 dez.

